# Ischaemic and Metabolic Treatment of Hepatic Tumours

**DOI:** 10.1155/1988/38715

**Published:** 1988

**Authors:** Bo Ahrén, Stig Bengmark

**Affiliations:** 1 Department of Surgery Lund University Lund Sweden; 2 Department of Surgery Lund University Lund 22185 Sweden

## Abstract

For treatment of malignancies, physical and metabolic differences between tumour cells and host cells have guided the development of new approaches. In this review, two new approaches to be used in the treatment of liver malignancies are outlined: ischaemic therapy and interferences with the glucose metabolism. Ischaemic therapy of liver malignancies has been used in different forms during the last 20 years: from ligation of the hepatic artery, embolization of the arterial tree, transient occlusion of the hepatic artery to the present day use of temporary, intermittent, transient hepatic arterial occlusion. The beneficial effect of ischaemic therapy on malignancies is supposed to depend on oxygen and nutritional deficiency, formation of oxygen-derived free radicals and loss of function in cellular enzymes. The tumour cells seem thereby to be more sensitive than the host cells. Also, ischaemia might potentiate the effect of cytotoxic drugs. Intereferencies with glucose metabolism might be directed either towards the exaggerated tumour glycolysis, for example by glucose analogues like 2-deoxy-glucose, or towards the exaggerated host gluconeogenesis, for example by hydrazine sulphate. These treatments result in reduction of the glucose availability in the intracellular glucose metabolism in the tumour cells and have experimentally been demonstrated to be correlated to reduced tumour growth. It is concluded that both these approaches, ischaemic therapy and manipulations with the glucose metabolism, seem promising for the future. What is needed now is research to clarify the mechanims behind the effects, to establish their full consequences, and to identify the clinical use of these treatments and their possible combinations.

## I. INTRODUCTION

For treatment of malignancies, physical and metabolic differences between tumour cells and host cells have guided the development of new approaches. For example, the greater sensitivity to cytotoxic agents in rapidly growing tumour cells compared to that in normal cells has made feasible the intense use of various cytotoxic drugs for treatment. Likewise, a sensitivity for radiation and hyperthermia greater in tumour cells than in normal cells has firmed the use of these models in cancer treatment. We will here focus attention on two other new approaches to be used in the treatment of malignancies: ischaemic therapy and interferences with the tumour glucose metabolism. For both these new approaches, differences in tumour versus host-cell function may explain the beneficial effects, and, furthermore, knowledge of these differences may be used when optimizing the therapy.

## II. HEPATIC DEARTERIALIZATION

Ischaemic therapy of liver malignancy was intitiated when it was realized that liver tumour cells preferentially are nourished by the arterial blood supply, whereas hepatocytes, on the contrary, are supplied mainly by portal blood[1,2]. Exceptions exist, however, since cells in small tumours and cells located peripherally in the tumours seem to be nourished also by the portal blood[3,4]. In the earlier phase of this development, ligation or embolization of the hepatic artery was performed[5–8]. This manipulation was found to reduce tumour growth, at least initially, but was also found to initiate collateral formation, which counteracts the aim of the ligation[9]. Therefore, a method of temporary dearterialization was developed: slings were introduced around the hepatic artery and removed after 16 hours[10]. This temporary dearterialization seemed to prevent collateral formation, but the technique was a one-shot treatment, which to be repeated necessitated a new laparatomy. Nevertheless, it was found to be beneficial for carcinoid tumours, with regard both to tumour volume reduction and to improvement of the carcinoid syndrome[11].

Recently, a new device allowing repeated hepatic arterial occlusion has been developed[12]. The occluder is connected to a subcutaneous injection port through which fluid may be injected, leading to hepatic artery occlusion (Figures 1 and 2). It is hoped that the repeatedly undertaken temporary dearterialization may prevent collateral formation and still retain the tumour reduction effect[13]. It has to be emphasized here that these arterial occlusion models all need the performance of an extensive devascularization procedure of the liver to make the hepatic artery the only supplier of arterial blood to the liver.

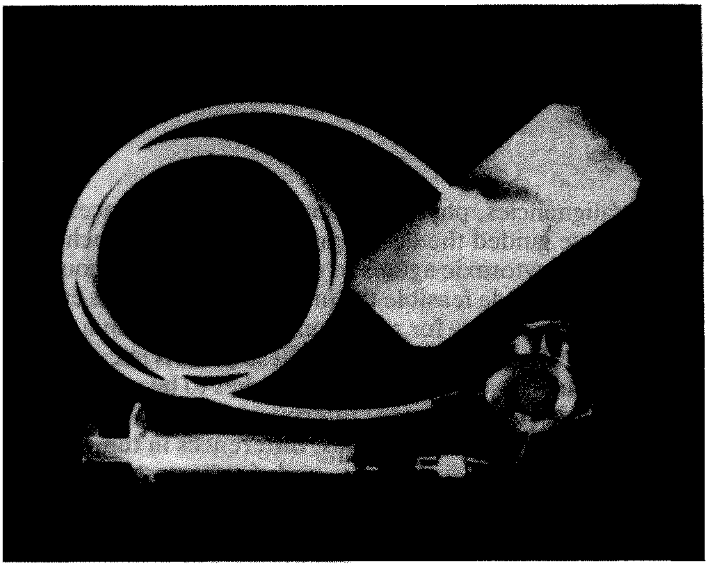

**Figure 1** Implantable device for intermittent, repeatedly undertaken dearterialization of the liver.

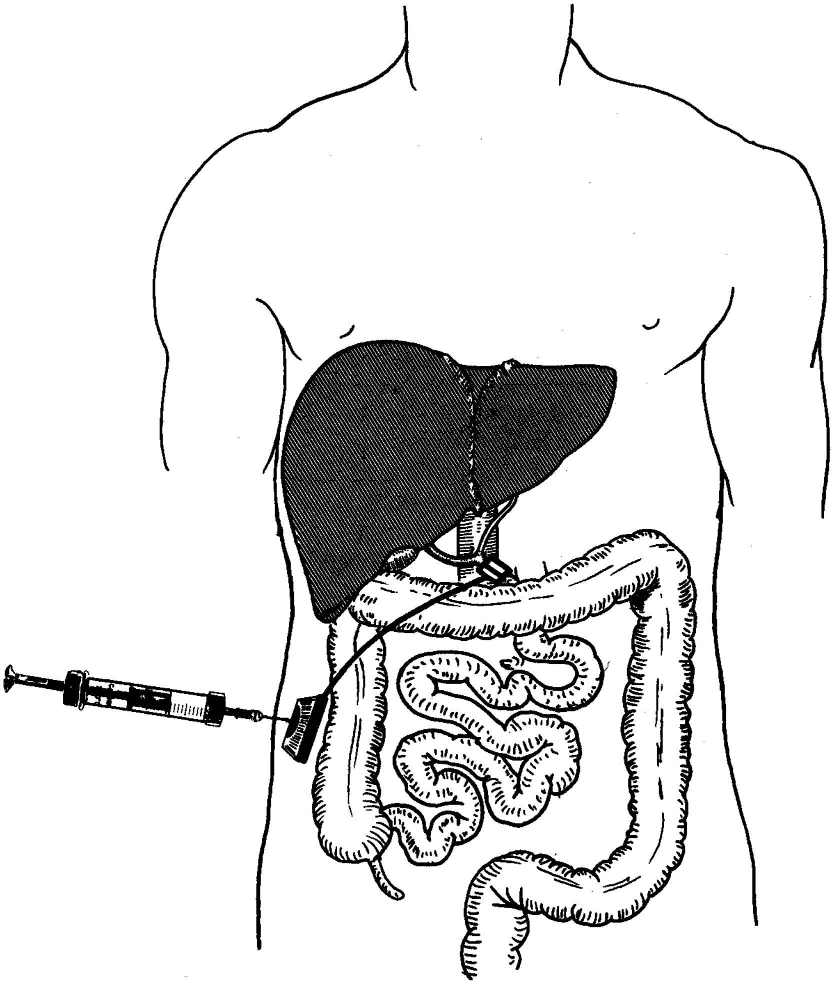

**Figure 2** The implantable occluder applied to the hepatic artery and connected to a port.

*Ischaemic Effects of Dearterialization*

A main effect of hepatic dearterialization is the resulting ischaemia. This eventually causes hypoxia in the tumours, as has been demonstrated by the clamping of experimental rodent tumours[14]. The oxygen lack halts cellular respiration, which subsequently results in cell damage. That oxygen lack indeed results in tumour necrosis has been demonstrated in a multicellular spheroid model of tumour microregions[15]. However, the same study also showed that necrosis may develop despite adequate oxygen supply, illustrating that other factors, such as glucose deprivation, acidosis or lactate accumulation, may also be of importance for necrosis development[15]. It is of interest to mention that small tumours might be more sensitive to ischaemia than large tumours, since they have a higher oxygen consumption rate[16]. Although tumour cells might thus be injured by the ischaemia occuring during dearterialization, the oxygen lack might also influence normal liver cells. A goal in this mode of treatment is to minimize the negative influence on the normal cells and still retain the beneficial effects on the tumours. Before reaching this goal, we have to analyze the characteristics and extent of ischaemia-induced injuries to the normal liver. It must, however, be mentioned that the tumours seem to be more sensitive to hypoxia than normal cells, probably because the rapid proliferation, notably of the tumour vascular endothelium[17].

Injured normal liver cells during and immediately after ischaemia have been demonstrated by the use of histochemical dye techniques[18]. Flocculent densities in the mitochondrial areas, as a sign of cell injury, have also been shown at 24 h after liver ischaemia in the rat[19]. Furthermore, the activity of the mitochondrial enzyme glutamate dehydrogenase is concomitantly decreased[19]. Moreover, after 3 h ischaemia of the rat liver, the activity of phospholipase A in the mitochondria has increased, which has resulted in accumulation of lysophospholipids and a subsequent injured control of the innner mitochondrial layer[20]. Also, after 5 h ischaemia of the rat liver, serum levels of the mitochondrial enzyme ornithine carbamyl transferase have increased[21]. Injuries to the mitochondria seem, therefore, to be a common manifestation of liver ischaemia. However, other hepatic functions might also be altered by ischaemic treatment. Thus, microvilli of the bile canaliculi have been demonstrated to disappear after 15 min ischaemia, and the activity of the plasma membrane enzyme 5'-mononucleotidase is lowered after 30 min ischaemia[22]. Lysosomal alterations have also been seen during hepatic ischaemia: the serum activity of the lysosomal enzyme β–glucosaminidase in the rat is lowered after 1 h hepatic ischaemia[23], and serum lysosomal enzyme activities in man are increased after hepatic dearterialization[24]. In addition, hypoxia and ischaemia in the perfused pig liver have been shown to impair the hepatic galactose elimination rate, inhibit ATP phosphorylation and increase the lactate output[25]. However, depite 80 min ischaemia, almost complete recovery occurred[25].

### *Other Effects of Dearterialization*

Besides the hypoxic-ischaemic effects of clamping procedures of the hepatic artery, these also seem to increase sensitivity to radiation and cytotoxic drugs, induce the formation of oxygen-derived free radicals, and restrict the nutritional supply to the tumours. These effects might also be of importance, perhaps as important as the ischaemic-hypoxic effects of dearterialization. That clamping enhances sensitivity to radiation has been demonstrated in rodent mammary tumours[14], and the potentiation of cytostatic drugs is exemplified by the anticancer agent mitomycin C, which has been demonstrated to be more cytotoxic to hypoxic tumour cells than to cells under normal oxygen tension[26]. Likewise, cisplatin seems to reduce the oxidative metabolism in polymorphonuclear cells, which might be indicative of a hypoxia-like action, which then will be further enhanced by hypoxia[27].

Ischaemic therapy has also been demonstrated to induce the formation of oxygen-derived free radicals[28]. These toxic substances (superoxide, hydrogen peroxide and hydroxyl radicals) are produced under hypoxic conditions and during reperfusion after hypoxia[29]. A mechanism behind their production is the reduction of hypoxanthine to xanthine by the enzyme xanthine dehydrogenase, which under hypoxic conditions functions as a xanthine oxidase[30]. Under normal conditions, protective mechanisms against this conversion exist, but these are impaired during hypoxia[31]. Consequently, xanthine oxidase inhibition by allopurinol has been demonstrated to prevent a long-standing reduction in protein synthesis after liver ischaemia in rats[32], and the radical scavenger disulfiram has been demonstrated to reduce the number of injured liver cells after 90 min ischaemia in rats[33]. Likewise, in one study, the scavenger glutathione was protective against rat liver ischaemia when intracellular calcium distribution, dye exclusion and membrane potential were investigated[34]. Hence, dearterialization might be deleterious to cells also through the production of oxygen-derived free radicals[35].

Clamping of the arterial supply reduces the nutritional supply to the cells. To what extent this may explain the cell injury and whether tumour cells are more vulnerable to this effect are questions that remain to be settled. However, it has been shown that protein synthesis in the liver is reduced by ischaemia[36–37], and it has also been demonstrated that glucose deprivation to tumour cells *in vitro* results in derangements of protein synthesis similar to those from hypoxia[38]. Furthermore, central tumour necrosis is induced not only by hypoxia but also by lack of glucose[15].

Thus, effects other than pure hypoxia seem to evolve by clamping and dearterialization. Of these, exaggerated sensitivity to cytotoxic treatment, superoxide production and deprivation of the nutritional supply seem to be most interesting in relation to tumour treatment. Combined treatment might be scheduled upon this knowledge.

### *Dearterialization: Conclusions*

As outlined in the discussion so far, the full consequences and exact mechanism of ischaemic therapy of liver malignancy are not established. It is clear, however, that not only hypoxic effects are induced, but also exaggerated sensitivities to cytotoxic substances, formation of oxygen-derived free radicals, and reduction of the nutritional supply. Experimentally, ischaemia has been shown to impair tumour growth[39] and, for future development and research, a small implantable device for repeated temporary hepatic dearterialization in the rat has been developed[40]. It will be used to study the consequences of hepatic ischaemia and to optimize treatment. Clinically, hepatic dearterialization used for 16 h has been shown to reduce carcinoid tumour growth[11]. Studies on the theoretically more optimal repeated temporary dearterialization are under way. Future research must establish the time dependency of effects on the tumour and the host cells to settle the optimal schedule for treatment. The optimal treatment should use the beneficial effect of tumour growth retardation but avoid ischaemic effects on normal liver cells. At present, a schedule of hepatic dearterialization 1 h twice daily is considered most proper and used in our department.

## III. MANIPULATION OF GLUCOSE METABOLISM

Several different types of tumour cells are characterized by deriving their energy mainly from the glycolytic pathway and thus they show a great dependency on glucose supply for their survival. This characteristic was recognized several decades ago[41] and has recently been reconfirmed by the demonstration that tumours exhibit high rates of glucose utilization and lactic acid production[42]. This high rate of glycolysis by tumours could be a mechanism behind the exaggerated glucose turnover rate in tumour patients, as shown by the glucose isotope tracer technique[43,44]. This abnormally high dependency on glucose is a potential difference between tumour and host cells to which intervention may be directed. Two different approaches may then be undertaken: reduction of the glycolytic activity and restrainment of the glucose supply.

### *Inhibition of Glycolysis*

The glycolytic pathway is the process in which glucose is converted to substrates for the tricarboxylic acid cycle and in which the energy of the glucose molecule is liberated as ATP. The pathway involves several different enzyme steps converting glucose-6-phosphate to pyruvic and lactic acid (Figure 3). The glycolytic flow can be inhibited by the substance 2-deoxyglucose, which is a glucose analogue (Figure 4). 2-Deoxyglucose inhibits both the glucose uptake into the cell, and the hexokinase that catalyzes the phosphorylation of glucose (i.e. the formation of glucose-6-phosphate)[45,46]. 2-Deoxyglucose is by itself converted to a 2-deoxyglucose-6-phosphate, but this substance is not isomerized to fructose-6-phosphate, and thus does not enter the glycolytic pathway further. 2-Deoxyglucose might also inhibit the conversion of glucose-6-phosphate to glucose-1-phosphate[47]. A marked reduction in glycolysis by 2-deoxyglucose has been demonstrated *in vitro* in leukaemic leukocytes and in HeLa cells[48,49]. Since tumour cells, as outlined above, seem more dependent on this pathway for their energy supply than other cells, 2-deoxyglucose might be used in the treatment of malignancies. This has been investigated experimentally in a few studies. In one study, the effects of 2-deoxyglucose on tumour growth in three different tumour models in the mouse were investigated: in a leukaemia, a transplantable carcinoma, and a mast cell tumour[50]. 2-Deoxyglucose was found to reduce tumour growth and induce a prolongation of the survival time[50]. Furthermore, a recent study on fibrosarcoma in the rat demonstrated that upon systemic administration 2-deoxyglucose reduced the tumour growth[51], and a recent

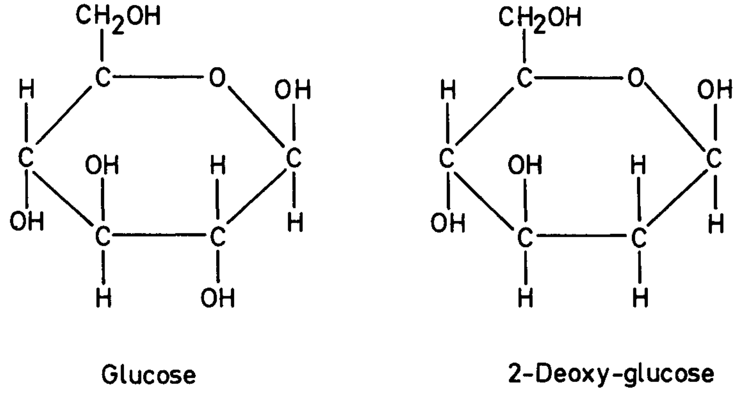

**Figure 4** The chemical structures of glucose and 2-deoxyglucose, illustrating that 2-deoxyglucose lacks the hydroxyl group in carbon position 2.

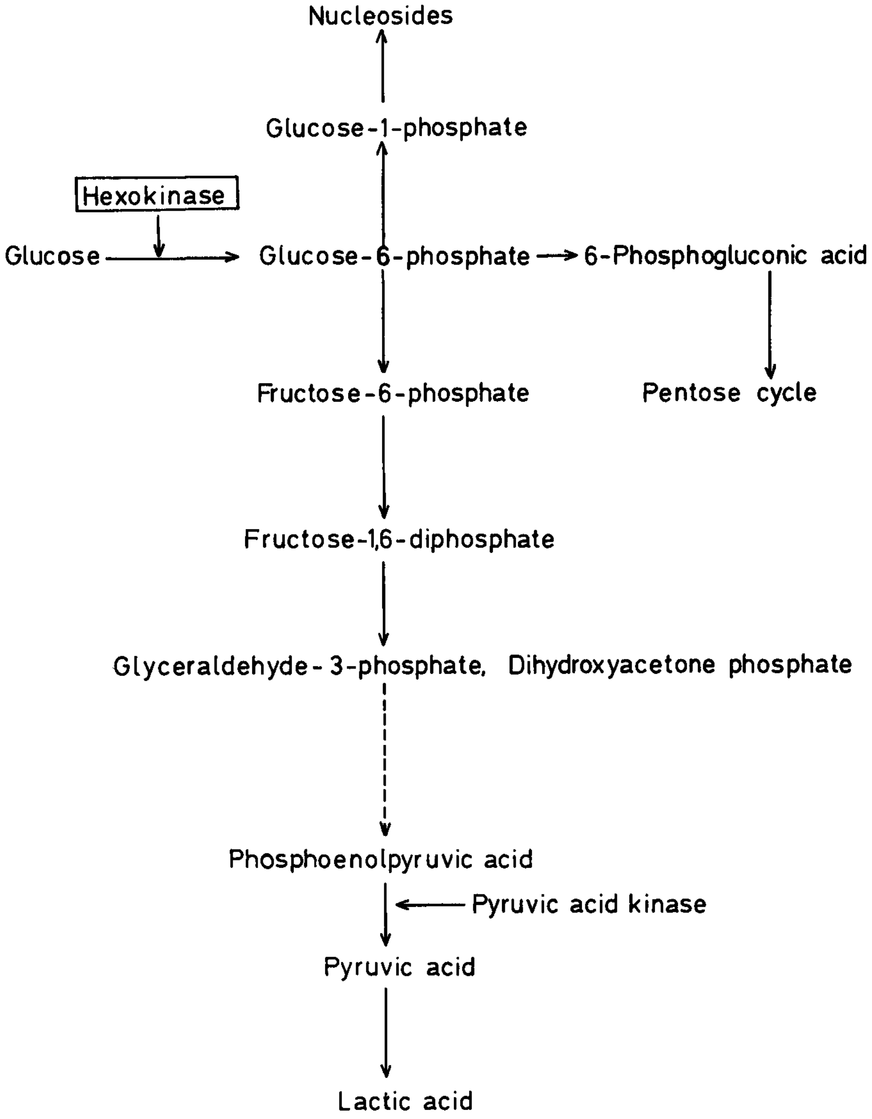

**Figure 3** A simplified outline of the intracellular metabolic fate of glucose, showing the glycolytic pathway in which glucose-6-phosphate is converted to pyruvic and lactic acid. 2-Deoxyglucose inhibits this pathway by inhibiting the cellular uptake of glucose and by inhibiting the enzyme hexokinase. Also illustrated in the figure is the enzyme pyruvic acid kinase, which in glycolysis converts phosphoenolpyruvic acid to pyruvic acid.

study on HeLa cells showed that 2-deoxyglucose at high-dose levels inhibited DNA repair after radiation[49]. However, in two other experimental tumour models in the mouse, a fibrosarcoma and a mammary adenocarcinoma, 2-deoxyglucose failed to alter tumour growth when administered at dose levels high enough to produce systemic toxicity[52]. This illustrates that different tumours might respond with different sensitivity to the inhibition of glycolysis, and that the systemic toxicity of glucose analogues might restrict their use. The toxicity depends on the neuroglycopenia, since a competition with glucose in the glycolytic pathway also occurs within the brain, which is strictly dependent on glucose for its metabolism. Neuroglycopenia causes neurological disturbances that may lead to seizures and death[52]. Also, neuroglycopenia induces reflex activation of the autonomic system, as evidenced by enhanced plasma levels of noradrenaline[53] and a cholinergically mediated stimulation of insulin secretion[54].

In conclusion, the approach of inhibiting the glycolytic pathway in tumours seems valid and might guide future research. What is needed is, first, to establish which malignancies are sensitive to this mode of treatment and, secondly, to develop inhibitors of glycolysis that exert effects on tumour metabolism without affecting neural glycolysis. Regional administration of glucose analogues might also reduce toxic systemic effects. Also, since hypoxic cells seem more influenced by glucose deprivation than normal cells, the combined use of glucose analogues and dearterialization procedures has to be investigated.

## *Inhibition of Gluconeogenesis*

The formation of glucose from non-carbohydrate precursors, mainly amino acids and lactate, is called gluconeogenesis (Figure 5). This pathway is often exaggerated in cancer patients because of the general catabolism, with an exaggerated production of amino acids from the protein breakdown, of glycerol from the massive lipid mobilization, and of lactate from the enhanced glycolytic pathway in the tumour[55]. This massive gluconeogenesis provides the tumour with amounts of glucose sufficient for the exaggerated glycolysis, and might be the pathway for the high energy expenditure in these patients and, possibly, the sign of cancer cachexia. It has

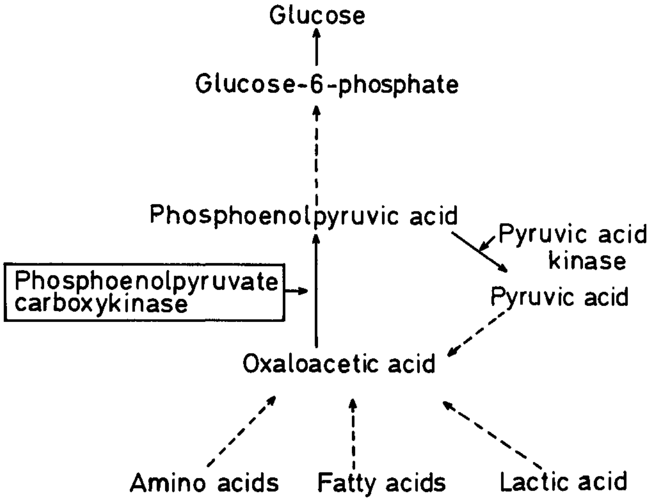

**Figure 5** A simplified outline of the metabolic pathways of gluconeogenesis, showing the conversion of amino acids, lipids and carbohydrates to glucose via the tricarboxylic acid cycle intermediates. Illustrated is the enzyme phosphoenolpyruvate carboxykinase, which converts oxaloacetate via pyruvic acid to phosphoenolpyruvic acid, and which is inhibited by hydrazine sulphate.

therefore been proposed that inhibition of the exaggerated gluconeogenesis would reverse, or at least diminish, cancer cachexia, and also by diminishing the provision of glucose to tumour cells inhibit tumour growth[56]. Theoretically, inhibition of gluconeogenesis could be achieved by inhibition of the enzyme phosphoenolpyruvate carboxykinase, i.e. the enzyme catalyzing the conversion of pyruvic acid to phosphoenolpyruvic acid[57]. By inhibition of this enzyme, gluconeogenesis would be selectively inhibited, without any inhibition of glycolysis, which lacks this enzyme in its pathway, since the conversion of phosphoenolpyruvic acid kinase to pyruvic acid in the glycolytic pathway is catalyzed by pyruvic acid kinase[57].

Hydrazine sulphate was found to be an agent that specifically inhibits phosphoenolpyruvate carboxykinase; the inhibition was found to be irreversible and readily obtained *in vivo* as well as *in vitro*[58]. Hydrazine sulphate belongs to a group of anti-cancer agents, of which the most well known is procarbazine[59]. Hydrazine sulphate has been tested experimentally and found to inhibit growth of a variety of experimental tumours, e.g. Walker 256 carcinosarcoma, Murphy–Sturm lymphosarcoma, B-16 melanoma, and fibrosarcoma and adenocarcinoma of the lung and breast in mice[60–63]. Subsequently, the drug was tested in a group of 84 patients with disseminated cancer and found to improve the patients subjectively in 70% and objectively in 17% within three months of treatment[64]. Furthermore, the effects of hydrazine sulphate were evaluated in a group of 233 patients with disseminated tumours during a period of 1–6 months, and the drug was found to reduce tumour growth in 50% of cases[65]. It also improved cancer cachexia[57,66]. A recent double-blind trial on 12 malnourished patients with lung cancer also showed that, after just 30 days of treatment, improvement of cancer cachexia was obtained[66]. However, negative results have also been seen: in a study on 25 patients with solid malignant tumours, no subjective or objective improvement was observed during a 4-week treatment period with hydrazine sulphate[67]. It might thus be concluded so far that inhibition of gluconeogenesis by the use of hydrazine sulphate improves metabolic and nutritional indices in patients with cancer cachexia, but it is too early to conclude whether this is combined with reduction in tumour growth and/or improved survival rates[67]. The studies with hydrazine sulphate, however, have illustrated the possibility of interfering with host metabolism, and this, perhaps together with manipulations directed towards tumour metabolism, might turn out a feasible direction for future research.

## IV. CONCLUSIONS

This review has focused on two new approaches for treatment of malignancies with special reference to hepatic malignancies: dearterialization and manipulation of the glucose metabolism. Both these approaches seem promising for the future. However, what is needed now is research undertaken to clarify the exact mechanisms behind the effects obtained by the different treatments, to establish their full consequences for both tumour and host cells, and to identify the clinical use of these treatments and their possible combinations.
